# Incidence and Risk Factors for Placenta Accreta/Increta/Percreta in the UK: A National Case-Control Study

**DOI:** 10.1371/journal.pone.0052893

**Published:** 2012-12-27

**Authors:** Kathryn E. Fitzpatrick, Susan Sellers, Patsy Spark, Jennifer J. Kurinczuk, Peter Brocklehurst, Marian Knight

**Affiliations:** 1 National Perinatal Epidemiology Unit, University of Oxford, Oxford, United Kingdom; 2 University Hospitals Bristol NHS Trust, Bristol, United Kingdom; 3 Institute for Women's Health, University College London, London, United Kingdom; Tehran University of Medical Sciences, Iran (Islamic Republic Of)

## Abstract

**Background:**

Placenta accreta/increta/percreta is associated with major pregnancy complications and is thought to be becoming more common. The aims of this study were to estimate the incidence of placenta accreta/increta/percreta in the UK and to investigate and quantify the associated risk factors.

**Methods:**

A national case-control study using the UK Obstetric Surveillance System was undertaken, including 134 women diagnosed with placenta accreta/increta/percreta between May 2010 and April 2011 and 256 control women.

**Results:**

The estimated incidence of placenta accreta/increta/percreta was 1.7 per 10,000 maternities overall; 577 per 10,000 in women with both a previous caesarean delivery and placenta praevia. Women who had a previous caesarean delivery (adjusted odds ratio (aOR) 14.41, 95%CI 5.63–36.85), other previous uterine surgery (aOR 3.40, 95%CI 1.30–8.91), an IVF pregnancy (aOR 32.13, 95%CI 2.03–509.23) and placenta praevia diagnosed antepartum (aOR 65.02, 95%CI 16.58–254.96) had raised odds of having placenta accreta/increta/percreta. There was also a raised odds of placenta accreta/increta/percreta associated with older maternal age in women without a previous caesarean delivery (aOR 1.30, 95%CI 1.13–1.50 for every one year increase in age).

**Conclusions:**

Women with both a prior caesarean delivery and placenta praevia have a high incidence of placenta accreta/increta/percreta. There is a need to maintain a high index of suspicion of abnormal placental invasion in such women and preparations for delivery should be made accordingly.

## Introduction

Abnormal placental adherence can be classified into three distinct conditions: placenta accreta, in which placental tissue invades the decidual surface of the myometrium; placenta increta, in which placental villi invade more deeply within the myometrium, and placenta percreta where chorionic villi penetrate through the uterine serosa and may invade surrounding organs such as the bladder. The presence of placenta accreta/increta/percreta is associated with major pregnancy complications [1], and is thought to be becoming more common [2], due to a number of factors including rising maternal age at delivery and an increasing proportion of deliveries by caesarean [3], [4]. This finding is of particular concern in the context of increasing rates of caesarean delivery and older maternal age at childbirth [5], [6]. However, the risk associated with these factors has not been quantified on a population basis in studies using robust clinical and pathological definitions.

The aims of this study were to estimate the national incidence of placenta accreta/increta/percreta in the UK and to investigate and quantify the associated risk factors in this population.

## Materials and Methods

### Study design & power

A national population-based case-control study was undertaken. Over the 12 month study period, we anticipated identifying 300 cases (based on an estimated incidence of 1 in 2500 [4]) and aimed to collect data on two controls for every case. This number of cases and controls would have given an estimated power of 80% at the 5% level of significance to detect odds ratios (ORs) between 1.6 and 2.3, assuming a prevalence range for potential risk factors of between 5% and 40% in the control women. The actual number of cases and controls identified during the study gave an estimated power of 80% at the 5% level of significance to detect ORs between 1.9 and 3.3, assuming the same risk factor prevalence levels.

### Case and control definition

Cases were all women in the UK identified as having placenta accreta/increta/percreta defined as either placenta accreta/increta/percreta diagnosed histologically following hysterectomy or post-mortem or an abnormally adherent placenta, requiring active management, including conservative approaches where the placenta is left in situ. Controls were defined as the two women who did not have placenta accreta/increta/percreta and delivered immediately before each case in the same hospital.

### Data collection

Cases were identified through the monthly mailing of the UK Obstetric Surveillance System (UKOSS) between 1^st^ May 2010 and 30^th^ April 2011. The UKOSS methods have been described in detail elsewhere [7]. Briefly, up to four nominated clinicians (anesthetists, midwives, obstetricians and risk managers) in each obstetrician-led maternity unit in the UK were sent a report card each month with a tick box to indicate the number of cases of placenta accreta/increta/percreta they had seen that month. They were asked to return all cards, including those with ‘nothing to report’, allowing participation to be monitored and confirmation of the denominator population for the study. Clinicians who reported a case were then sent a data collection form requesting further details to confirm the case definition and describe potential risk factors, management and outcomes. Reporting clinicians were also asked to select and complete a data collection form for two controls, identified as the two women meeting the control definition. Up to five reminders were sent if complete forms were not returned. All data requested were anonymous. On receipt of data collection forms, the data were double entered into a customised database. Cases were checked to confirm that they met the case definition and controls were checked to ensure they had been selected correctly. Duplicate reports were identified by comparing the woman's year of birth and expected date of delivery. Where data were missing or a data validity check flagged up a problem, reporting clinicians were contacted and asked to provide or check the information.

### Statistical analysis

The overall incidence with 95% confidence intervals (CIs) of placenta accreta/increta/percreta was calculated using the most recently available national birth data (2010 [8], [9], [10]) as a proxy denominator for the number of maternities during the study period. Denominator data to calculate the incidence and 95% CIs of placenta accreta/increta/percreta in women with and without a previous caesarean delivery and in women with and without placenta praevia diagnosed prior to delivery, were estimated using the proportions of women in these various categories observed in the control women together with the most recently available birth data. To calculate the incidence and 95% CIs of placenta accreta/increta/percreta in women with a previous caesarean with and without placenta praevia diagnosed prior to delivery, we used an estimate of the incidence of placenta praevia in women with a previous caesarean delivery (1.2%), derived from a recent systematic review [11].

Potential risk factors for placenta accreta/increta/percreta were investigated by comparing the women with accreta/increta/percreta to the control group of women using unconditional logistic regression to estimate ORs and 95% CIs. A full regression model was developed by including both explanatory and potential confounding factors in a core model if there was a pre-existing hypothesis or evidence to suggest they were causally related to placenta accreta/increta/percreta. Factors with a high proportion of missing data (>20%) were omitted from the full model where there was no evidence (p>0.20) in the univariate analysis that they were associated with accreta/increta/percreta. Continuous explanatory and potential confounding variables were tested for departure from linearity by the addition of first-order fractional polynomials to the model and subsequent likelihood ratio testing. Where there was evidence for non-linearity, continuous variables were presented and treated as categorical in the analysis. Where there was no evidence of departure from linearity, continuous variables are presented as categorical for ease of interpretation, but have been treated as continuous linear terms when adjusting for them in the analysis. Plausible interactions were tested in the full regression model by the addition of interaction terms and subsequent likelihood ratio testing on removal, with a p-value of <0.01 considered evidence of significant interaction to account for multiple testing. All analyses were carried out using STATA v11 software.

### Ethics statement

This study was approved by the London Research Ethics Committee (ref 10/H0717/20).

## Results

### Incidence

**Figure 1:**
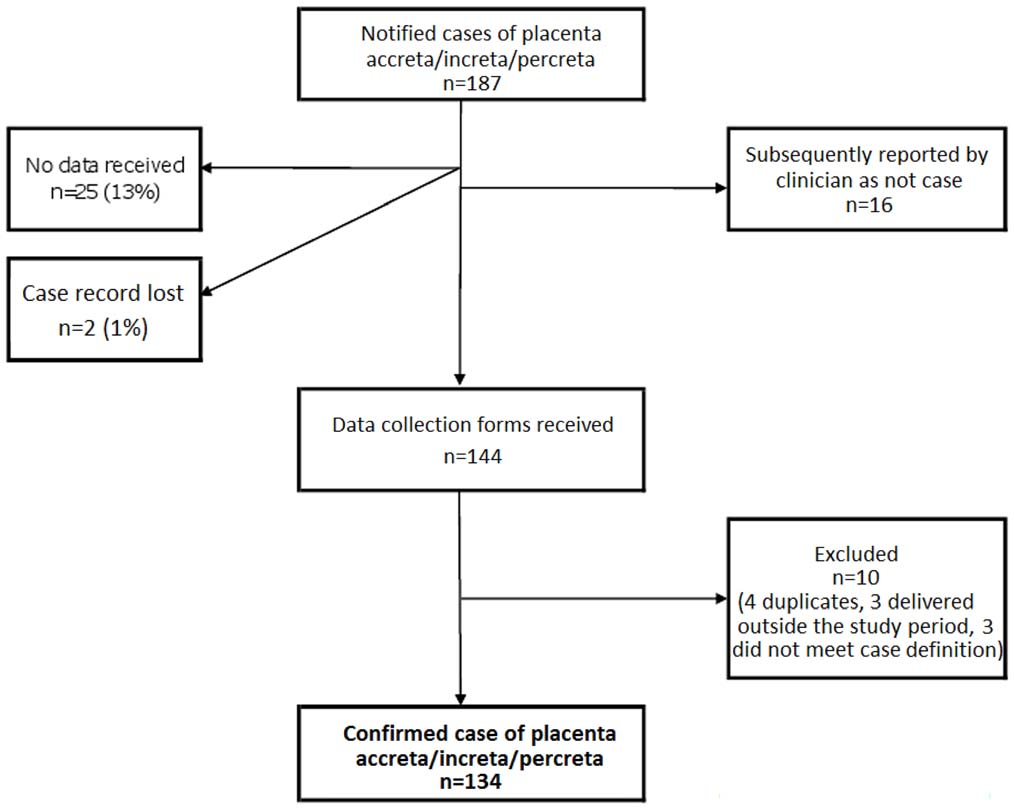
Case reporting and completeness of data collection.

All 221 UK hospitals with obstetrician-led maternity units participated in UKOSS during the study period, representing 100% participation. Of the 187 notified cases of placenta accreta/increta/percreta, 16 were subsequently reported by clinicians as not cases. Data collection forms were received for 144 (84%) of the remaining notified cases (Figure 1) and data were obtained for 256 controls.There was a total of 134 confirmed cases of placenta accreta/increta/percreta in an estimated 798634 maternities [8], [9], [10], representing an estimated incidence of 1.7 per 10,000 maternities (95% CI 1.4 to 2.0). Table 1 shows the estimated incidence of accreta/increta/percreta for various categories of women; incidence estimates range from an estimated 1 in 33, 000 for women without a previous caesarean delivery to an estimated 1 in 20 for women with at least one previous caesarean delivery and placenta praevia diagnosed prior to delivery.

**Table 1:** Estimated incidence of placenta accreta/increta/percreta for different categories of women.

Category¥	Number of women with placenta accreta/increta/percreta	Estimated number of maternities	Estimated incidence of placenta accreta/increta/percreta (95% CI) per 10,000 maternities
Women without a previous caesarean delivery	21	678839	0.3 (0.2–0.5)
Women with at least one previous caesarean delivery	113	119795	9 (8–11)
Women without placenta praevia diagnosed prior to delivery	47	790648	0.6 (0.4–0.8)
Women with placenta praevia diagnosed prior to delivery	86	7986	108 (86–133)
Women with at least one previous caesarean delivery but without placenta praevia diagnosed prior to delivery	30	118357	3 (2–4)
Women with at least one previous caesarean delivery and placenta praevia diagnosed prior to delivery	83	1438	577 (462–711)

¥ Categories are not mutually exclusive.

### Risk factors

Table 2 shows the characteristics of the women with placenta accreta/increta/percreta compared to the control women. The odds of having placenta accreta/increta/percreta rose with increasing maternal age (adjusted OR (aOR) 1.15, 95% CI 1.06 to 1.24 for every one year increase in age; presented in Table 2 as a binary variable for ease of interpretation). The odds of having placenta accreta/increta/percreta were also raised in women who had a previous caesarean delivery (aOR 14.41, 95% CI 5.63 to 36.85). There did not appear to be a linear association between placenta accreta/increta/percreta and number of previous caesarean deliveries, with women who had two or more previous caesareans having similar odds of placenta accreta/increta/percreta to women with one previous caesarean (p = 0.810). However, the power of this analysis was limited by the small number of women with two or more previous caesarean deliveries (22 case and nine control women had two previous caesarean deliveries; 20 case and two control women had three previous caesarean deliveries; and eight case and one control women had four or more previous caesarean deliveries). There was evidence of an interaction between age and previous caesarean delivery; the raised odds associated with older maternal age was only apparent in women without a previous caesarean delivery (aOR 1.30, 95% CI 1.13 to 1.50 for every one year increase in age in women without a previous caesarean; aOR 1.03, 95% CI 0.92 to 1.15 for every one year increase in age in women with a previous caesarean).

**Table 2:** Risk factors for placenta accreta/increta/percreta.

Risk factor	Number (%)* of cases (n = 134)	Number (%)* of controls (n = 256)	Unadjusted OR (95% CI)	P-value	Adjusted‡ OR (95% CI)	P-value
*Sociodemographic factors*						
**Age (years)**						
Less than 35	57 (43)	194 (76)	1		1	
35 or older	77 (57)	61 (24)	4.3 (2.75–6.72)	<0.0001	3.48 (1.52–7.96)	0.0032
**Ethnic group**						
White	99 (74)	210 (83)	1		1	
Non-white	34 (26)	44 (17)	1.64 (0.99–2.72)	0.0563	0.66 (0.23–1.85)	0.4251
**Socio-economic group**						
Managerial & professional occupations	36 (34)	63 (31)	1			
Other	70 (66)	143 (69)	0.86 (0.52–1.41)	0.5437		
**Body mass index at booking (Kg/m^2^)**						
Less than 25	60 (46)	135 (54)	1		1	
25–29.9	42 (32)	65 (26)	1.45 (0.89–2.38)	0.1368	0.98 (0.36–2.71)	0.972
30 or more	29 (22)	49 (20)	1.33 (0.77–2.31)	0.3081	0.57 (0.18–1.79)	0.3366
**Smoking status**						
Never/ex smoker	107 (80)	213 (85)	1		1	
Smoked during pregnancy	26 (20)	39 (15)	1.33 (0.77–2.30)	0.3114	0.53 (0.15–1.84)	0.3186
*Previous obstetric & medical history*						
**Parity**						
0	12 (9)	106 (41)	1		1	
1	39 (29)	84 (33)	4.1 (2.02–8.32)	0.0001	0.59 (0.15–2.39)	0.4638
2 or more	83 (62)	66 (26)	11.11 (5.63–21.90)	<0.0001	0.9 (0.22–3.64)	0.8826
**Number of previous caesarean deliveries**						
0	21 (16)	218 (85)	1		1	
1	63 (47)	25 (10)	26.16 (13.73–49.83)	<0.0001	14 (5.31–36.93)	<0.0001
2 or more	50 (37)	12 (5)	43.25 (19.97–93.70)	<0.0001	16.31 (4.09–64.99)	0.0001
**Previous caesarean uterine incision type(s)**						
All low transverse incisions	105 (99)	34 (100)				
Any non-low transverse incisions	1 (1)	0 (0)				
**Other previous uterine surgery** ¥						
No	94 (71)	224 (88)	1		1	
Yes	39 (29)	31 (12)	3 (1.77–5.09)	<0.0001	3.4 (1.30–8.91)	0.0128
**Previous uterine perforation**						
No	133 (100)	254 (100)				
Yes	0 (0)	0 (0)				
*Current pregnancy*						
**Twin pregnancy**						
No	130 (97)	252 (98)	1		1	
Yes	4 (3)	4 (2)	1.94 (0.48–7.88)	0.3548	2.99 (0.28–32.42)	0.3685
**Interval between last caesarean section and last menstrual period (months)**						
24 or more	61 (55)	21 (58)	1			
12–23	32 (29)	10 (28)	1.1 (0.46–2.62)	0.8266		
Less than 12	17 (15)	5 (14)	1.17 (0.38–3.56)	0.7817		
**Placenta praevia diagnosed prior to delivery**						
No	47 (35)	253 (99)	1		1	
Yes	86 (65)	3 (1)	154.31 (46.83–508.51)	<0.0001	65.02 (16.58–254.96)	<0.0001
**Female infant(s)**						
No	52 (40)	136 (53)	1		1	
Yes	78 (60)	119 (47)	1.71 (1.12–2.63)	0.0137	1.25 (0.55–2.81)	0.593
**IVF pregnancy**						
No	126 (96)	253 (100)	1		1	
Yes	5 (4)	1 (0)	10.04 (1.16–86.85)	0.0362	32.13 (2.03–509.23)	0.0138
**Pregnancy induced hypertension or pre-eclampsia**						
No	127 (97)	247 (97)	1		1	
Yes	4 (3)	7 (3)	1.11 (0.32–3.87)	0.8682	3.06 (0.48–19.53)	0.2361

* Percentage of individuals with complete data.

¥ Includes myomectomy, dilation & curettage, surgical termination of pregnancy, evacuation of retained products of conception & manual removal of placenta.

‡ Adjusted for all factors in the table apart from socio-economic group, previous caesarean uterine incision type(s), previous uterine perforation and interval between last caesarean section and last menstrual period. When adjusting for age, BMI and parity, these variables have been treated as a continuous linear term in the analysis.

Women who had other previous uterine surgery such as myomectomy also had an increased odds of having placenta accreta/increta/percreta (aOR 3.40, 95% CI 1.30 to 8.91), as did women who had an IVF pregnancy (aOR 32.13, 95% CI 2.03 to 509.23). The odds of placenta accreta/increta/percreta were also raised in women who had placenta praevia diagnosed antenatally (aOR 65.02, 16.58 to 254.96). Most of the case women diagnosed with placenta praevia had major praevia (66/79, 84% grade 4), whilst the small number of control women diagnosed with placenta praevia all had minor praevia (2/2, 100% grade 1 or 2). As placenta praevia may be on the causal pathway between previous caesarean delivery and placenta accreta/increta/percreta a further analysis was performed in which placenta praevia was removed from the multivariable model. Removing placenta praevia more than doubled the odds associated with previous caesarean delivery (aOR 36.45, 95% CI 16.62 to 79.95), although there was still no evidence of a linear association between placenta accreta/increta/percreta and number of previous caesarean deliveries; none of the other variables in the model were significantly altered (data not shown).

## Discussion

This study demonstrates the rarity of placenta accreta/increta/percreta using a robust clinical and pathological definition, estimating the incidence in the UK to be 1.7 per 10,000 maternities overall. However, the incidence is considerably higher in women with both a previous caesarean delivery and placenta praevia, occurring in around one in every twenty such women. In the absence of a completely sensitive and specific antenatal diagnostic technique [12], this highlights the importance of having a high index of suspicion of abnormal placental invasion and making preparations for delivery accordingly in this group of women.

Our incidence figures are lower than those quoted in recent studies. For example, the overall reported incidence of placenta accreta/increta/percreta ranges from four per 10,000 deliveries (based on a US study which identified 62 cases) [4] to as high as 90 per 10,000 deliveries (based on 310 cases identified during a study in Israel) [13]. A number of factors may account for these differences. There is no consensus clinical definition for placenta accreta/increta/percreta, the gold standard being pathological diagnosis, which is clearly only applicable to cases where hysterectomy has been performed. We used a combined clinical and pathological definition designed to capture severe cases which required active management but in which the uterus was successfully conserved as well as cases resulting in hysterectomy and in which a pathological diagnosis was available. Other studies, particularly those relying on routinely coded data, may not have used such a rigorous clinical definition, including false positive cases.

Other methodological differences may account for our lower incidence estimates. The existing literature consists predominately of studies undertaken using retrospective review of medical records over a number of years in a single or small number of hospitals. One limitation of such studies is their hospital-based nature makes them prone to overestimating the incidence, as high risk and emergency cases tend to be referred into such hospitals from surrounding sites. Although estimated, we are confident that our denominator data accurately reflects the true denominator: the total number of maternities in the UK over the study period was estimated using the most recently available national birth data (2010 [8], [9], [10]), covering much of the same time period as our study; and our control group of women, who are comparable in characteristic to the available national data on women giving birth in the UK, were used to estimate the proportion of women in the UK with and without a previous caesarean section and the proportion with and without placenta praevia. Although we used data from a recent systematic review [11] to estimate the rate of placenta praevia in women with a previous caesarean section in the UK, to our knowledge, this is the best available current estimate in a developed country setting, being derived from a number of studies rated as good or fair quality.

Despite the presence of several reporting clinicians in each hospital and the active, prospective nature of the UKOSS reporting system with the requirement of participating hospitals to return a report card every month regardless of whether they had cases to report, we cannot exclude the possibility that our lower incidence estimates are due to underreporting of cases. We had no additional sources of data to check our case ascertainment, although previous studies using UKOSS have suggested high rates of ascertainment [14], [15].

Another explanation for the differences is that they may reflect a true variance in the rates and patterns of risk factors for the condition between the study populations. Placenta praevia [3], [4], [13], [16], previous caesarean delivery [3], [4], [13], [17], [18], other previous uterine surgery [13], [19], [20], multiparity [13], [20], advanced maternal age [3], [4], [13], [16], hypertensive disorders [17], smoking [17], IVF pregnancy [21] and a female fetus [22] are all factors that have previously been suggested as being associated with a higher risk of placenta accreta/increta/percreta. Our study investigated each of these factors and found that the risk of placenta accreta/increta/percreta was independently increased in women with a previous caesarean delivery; in women with other previous uterine surgery; in women with an IVF pregnancy and in women with placenta praevia diagnosed antepartum. The rise in odds associated with previous caesarean delivery observed when placenta praevia was removed from the multivariable model suggests that placenta praevia may partially mediate the association between previous caesarean delivery and placenta accrete/increta/percreta. There was also an increased risk of placenta accreta/increta/percreta associated with older maternal age in women without a previous caesarean delivery.

A few studies conducted outside of the UK have reported an increased incidence of placenta accreta/increta/percreta with increasing number of previous caesarean deliveries [4], [18], [23], [24]. Furthermore, a recent UK study that used UKOSS reported a rate of placenta accreta/increta/percreta of around 1,400 per 10,000 in women undergoing their fifth or greater caesarean section [25], considerably higher than our estimate of 9 per 10,000 in women with at least one previous caesarean delivery. Although this suggests that the risk of placenta accreta/increta/percreta may increase with the number of previous caesarean deliveries, we did not find a linear association between the condition and number of previous caesarean deliveries; our study suggests that women who have two or more previous caesarean deliveries have a similar risk of placenta accreta/increta/percreta to the women with one previous caesarean delivery. However, it is possible that our study lacked sufficient power to detect a difference due to the relatively small number of women with multiple previous caesarean deliveries.

### Conclusions

The risk of placenta accreta/increta/percreta appears to be raised in women who have a previous caesarean delivery, other previous uterine surgery, an IVF pregnancy and placenta praevia diagnosed antepartum. There was also an increased risk of placenta accreta/increta/percreta associated with older maternal age in women without a previous caesarean delivery. Although clinically significant placenta accreta/increta/percreta is uncommon overall, the high incidence of the condition in women with a prior caesarean delivery as well as placenta praevia highlights the importance of maintaining a high index of suspicion of abnormal placental invasion and making preparations for delivery accordingly in this group of women.
